# Niacin alleviates cerebral microvascular endothelial cell apoptosis induced by hypervirulent *Klebsiella Pneumoniae* by reducing the accumulation of reactive oxygen species

**DOI:** 10.3389/fcimb.2026.1867024

**Published:** 2026-07-10

**Authors:** Yinghai Zhao, Mubin Wang, Xing Che

**Affiliations:** 1 Department of Vascular Surgery, The Affiliated Taizhou People’s Hospital of Nanjing Medical University, Taizhou School of Clinical Medicine, Nanjing Medical University, Taizhou, Jiangsu, China; 2 Department of General Surgery, Jingjiang People’s Hospital Affiliated to Yangzhou University, Jingjiang, Taizhou, Jiangsu, China

**Keywords:** apoptosis, hypervirulent *Klebsiella pneumoniae*, meningitis, niacin, ROS

## Abstract

**Introduction:**

Hypervirulent *Klebsiella pneumoniae* (hv*K*p) is an important pathogen causing central nervous system infections, including meningitis; however, the mechanisms by which it disrupts the blood-brain barrier remain unclear. Niacin, a vitamin B3 compound related to NAD+ metabolism, has the potential to regulate redox homeostasis. This study aimed to investigate the mechanism of hv*K*p-induced injury in human cerebral microvascular endothelial cell (HCMEC) and to evaluate the protective effects of niacin.

**Methods:**

A GFP-labeled hv*K*p ATCC 43816 strain was used to establish an HCMEC infection model, and an hv*K*p mouse meningitis model was also constructed. Live-cell imaging was performed to monitor infection dynamics. Flow cytometry was used to assess infection rate, reactive oxygen species (ROS) levels, and Annexin V-FITC/PI-labeled apoptosis, while Western blotting was used to analyze apoptosis-related pathways. The experimental groups included a control group, an hv*K*p infection group, an hv*K*p plus niacin treatment group, and a niacin pretreatment group.

**Results:**

hv*K*p caused time- and dose-dependent injury to HCMEC, characterized by intracellular proliferation followed by apoptosis induction. Mechanistically, hv*K*p promoted mitochondrial ROS accumulation and activated the p53-associated mitochondrial apoptotic pathway. Niacin reduced ROS levels, inhibited apoptosis, and alleviated HCMEC injury. *In vivo*, niacin preserved blood-brain barrier integrity, reduced central nervous system inflammation and hv*K*p colonization, and improved survival in infected mice.

**Discussion:**

hv*K*p damages cerebral microvascular endothelial cells and disrupts the blood-brain barrier through the ROS-p53-mitochondrial apoptosis axis. Niacin exerts protective effects by regulating redox homeostasis, suggesting that it may serve as a host-directed adjunctive therapeutic strategy for hv*K*p-associated central nervous system infections.

## 1 Introduction

*Klebsiella pneumoniae (Kp*) is a globally prevalent opportunistic pathogen causing diseases such as pneumonia and bacteremia (Magill et al., 2018; Wyres et al., 2020). In recent years, a hv*Kp* with distinct phenotypic and genotypic characteristics has emerged as a significant pathogen in both community-acquired and hospital-acquired infections (Gu et al., 2018; Russo and Marr, 2019). hv*Kp* can infect healthy individuals across all age groups, and strains exhibiting high virulence and multidrug resistance have emerged in recent years (Han et al., 2022; Lan et al., 2021). Notably, hv*Kp* has become a major cause of bacterial meningitis and brain abscesses (Carrie et al., 2019; Huang et al., 2022; Zhao et al., 2022). In South Korea, hv*Kp* ranks as the third most common pathogen in community-acquired bacterial meningitis (Moon et al., 2010); In Taiwan, China, this bacterium accounts for approximately 25%–40% of adult bacterial meningitis cases, with associated mortality rates reaching 48.5%–66% (Fang et al., 2000; Ku et al., 2017; Siu et al., 2012). Given the multidrug resistance exhibited by hv*Kp* in recent years, the use of natural metabolites to augment antibiotic therapy has become a major research trend in the treatment of hv*Kp*.

Niacin, also known as vitamin B3, is the classic drug for treating pellagra. Within the body, niacin undergoes a series of biochemical reactions to convert into nicotinamide adenine dinucleotide (NAD). Subsequently, NAD is absorbed by cells via diffusion or transport proteins, playing a central role in redox reactions (Zapata-Perez et al., 2021). NAD exists in oxidized (NAD^+^) and reduced (NADH) forms, regulating cell death through multiple mechanisms (Doroftei et al., 2020; Jaeschke et al., 1992; Suchard and Savulescu, 2022). Moderate niacin supplementation has demonstrated therapeutic effects against cardiovascular disease, cancer, and age-related disorders (Freese and Lysne, 2023). Notably, the central nervous system is the tissue with the highest NAD^+^ consumption (Garg et al., 2017), and niacin can directly cross the blood-brain barrier to exert regulatory effects (Jiang et al., 2024). Furthermore, niacin is recognized as a key regulator of neuronal development and survival, holding broad application prospects in neurodegenerative diseases (Gasperi et al., 2019). Recent studies indicate that exogenous supplementation with β-nicotinamide mononucleotide (NMN, a NAD precursor) effectively prevents hv*Kp*-induced liver abscess formation (Cao et al., 2025). Does niacin also have preventive or therapeutic effects against hv*Kp*-induced meningitis? However, no studies have yet been reported in this area.

The blood-brain barrier serves as the central nervous system’s most critical defense against pathogen invasion. However, current research on hv*Kp* meningitis remains largely case-report based, with its specific mechanisms for breaching the blood-brain barrier and infecting the central nervous system still unclear. The blood-brain barrier is primarily composed of BMECs and astrocytes, with HMEC serving as the core component for its structural integrity and functional execution (Kadry et al., 2020). This study systematically analyzed the spatiotemporal dynamics and pathogenic mechanisms of hv*Kp* infection in HCMEC using an *in vitro* infection model and an *in vivo* hv*Kp* mouse meningitis model. It elucidated the protective effects and molecular mechanisms of exogenous niacin against hv*Kp*-induced endothelial cell apoptosis, oxidative stress damage, and blood-brain barrier disruption, thereby providing new host-directed therapeutic strategies for the clinical prevention and treatment of central nervous system infections caused by hv*Kp.*

## 2 Materials and methods

### 2.1 Bacterial strains and culture conditions

The hv*Kp* (ATCC 43816) strain was obtained and is maintained at the Anhui Provincial Bacterial Antimicrobial Resistance Surveillance Center laboratory. ATCC 43816 was cultured in Mueller-Hinton broth at 37 °C with shaking at 220 rpm overnight. The culture was diluted 1:100 with 5 mL of fresh medium and further cultured for 2 hours until it reached the logarithmic growth phase. According to the 0.5 McFarland standard, the bacterial CFU count was 1.5 × 10^8^ CFU/mL.

GFP-labeled ATCC 43816 carries a chloramphenicol resistance gene and was screened by adding 25 μg/mL of chloramphenicol to Mueller-Hinton broth.

### 2.2 Cell infection model

Seed 1 × 10^5^ HCMEC/D3 (ATCC; GNHu68) per well in a 6-well plate and culture until 80% confluence is reached. Discard the old medium, wash twice with sterile PBS, and add 2 mL of serum-free DMEM medium. Add GFP-labeled ATCC-43816 to each well at MOIs of 100, 50, 10, 5, 1, and 0, respectively. Incubate at 37 °C for various durations, then wash three times with PBS to remove unadhered bacteria. Digest the cells with trypsin to obtain a single-cell suspension. Assess the infection rate of ATCC-43816 by measuring the mean fluorescence intensity (MFI) at different MOIs via flow cytometry.

Four treatment groups for niacin: CON: untreated; hv*Kp* (MOI = 100, 6 h): Infection with hv*Kp* at MOI = 100 for 6 h; hv*Kp* + niacin (100 μM, 24 h): Cells were treated simultaneously with hv*Kp* at MOI = 100 and 100 μM niacin; hv*Kp* was removed 6 h after infection; pre-niacin (100 μM, 6 h) + hv*Kp*: Cells were pretreated with 100 μM niacin for 6 h, followed by infection with hv*Kp* (MOI = 100) for 6 h.

### 2.3 Live-cell imaging (live-cell workstation)

Seed HCMEC in a confocal culture dish (cat. 801001, NEST). Once the cells have attached, infect them with GFP-labeled ATCC-43816 at the corresponding MOI. Place the culture dish in the live-cell workstation and set the culture conditions to 37 °C, 5% CO_2_, and humidity > 95%. Acquire phase-contrast and fluorescence images every 2 hours for a total of 6 hours. Use the accompanying software to analyze changes in cell morphology and fluorescence intensity dynamics.

Treated HCMEC were digested with 0.25% trypsin (C25200056, Gibco) to form a single-cell suspension, washed twice with sterile PBS, and resuspended to a concentration of 1 × 10^6^ cells/mL. Add DCFH-DA probe (S0033S, Beyotime) to a final concentration of 10 μmol/L, and incubate at 37 °C in the dark for 30 minutes. Wash twice with PBS, then measure the mean fluorescence intensity using a flow cytometer (488 nm excitation, 525 nm emission). Record raw data from 10,000 cells per group.

### 2.4 Apoptosis assay (Annexin V-FITC/PI double staining method)

Treat the cells with trypsin to obtain a single-cell suspension, then wash once with sterile PBS. Resuspend the cells in 1× Annexin V-FITC Binding Buffer (C1062S, Beyotime) to a final concentration of 1 × 10^6^ cells/mL. Add 5 μL of Annexin V-FITC and 5 μL of PI to 195 μL of the cell suspension, and incubate at room temperature in the dark for 15 minutes. Simultaneously, prepare blank tubes and single-staining tubes with FITC or PI for voltage and compensation adjustments. Analyze the samples using a flow cytometer, collecting raw data from 10,000 cells per group.

### 2.5 Western blotting

Lysed the treated cells on ice for 30 minutes using RIPA lysis buffer (P0013B, Beyotime) containing PMSF and phosphatase inhibitors, then centrifuge at 12,000 g for 15 minutes and collect the supernatant. Determine the protein concentration of the sample using the BCA method, then add SDS loading buffer (P0015, Beyotime) in a 1:4 ratio. Heat at 95 °C for 10 minutes to completely denature the proteins. Load 20 μg of protein sample onto an SDS-PAGE gel and transfer the bands to a PVDF membrane (ISEQ00010, Merck). Block with 5% skim milk powder (P0216, Beyotime) at room temperature for 1 hour, then incubate with the primary antibody overnight at 4 °C. Unbound primary antibody was washed away with TBST, followed by incubation with HRP-conjugated secondary antibody at room temperature for 1 hour. Finally, development was performed using ECL chemiluminescent substrate. Band gray values were analyzed using ImageJ 1.54p, and the relative expression of the target protein was calculated using β-actin as an internal control.

The primary antibodies used were as follows: phospho-p53 (1:1000, 67826-1-RR), cleaved caspase-3 (1:1000, 82707-13-RR), Cleaved PARP1 (1:1000, 60555-1-RR), Bax (1:1000, 50599-2-RR), Bcl2 (1:1000, 80313-1-RR), SOD2 (1:1000, 24127-1-AP), Cytochrome c (1:1000, 10993-1-AP), β-actin (1:5000, 66009-1-lg).

### 2.6 TUNEL fluorescent staining

Cells were seeded on cell culture slides and treated once confluence reached 50%. The cells were divided into four groups: CON, hv*Kp* (MOI = 100, 6 h), hv*Kp* + niacin (100 μM, 24h), and pre-niacin (100 μM, 6h) + hv*Kp*. They were fixed with 4% paraformaldehyde for 30 minutes and permeabilized with 0.3% Triton X-100 for 10 minutes. Reaction solution was added according to the TUNEL kit instructions (C1089, Beyotime), and the samples were incubated at 37 °C in the dark for 60 minutes. Stain nuclei with DAPI for 5 min, then mount with an anti-fade mounting medium. Observe and capture images using a laser confocal microscope, and use ImageJ software to quantitatively analyze the average fluorescence intensity of TUNEL-positive cells.

### 2.7 Assessment of drug synergy using the microplate assay

Prepare serial two-fold dilutions of gentamicin (GM, G1397, Merck) or meropenem (1392454, Merck) with niacin (N4126, Merck) in a 96-well microplate, adjusting the final volume in each well to 50 μL of MHA broth. Culture ATCC 43816 to the logarithmic growth phase and adjust the bacterial concentration to 1.0 × 10^6^ CFU/mL using fresh MHA broth. Add 50 μL of bacterial suspension to each well, resulting in a final volume of 100 μL per well (final concentration of ATCC 43816: 5 × 10^5^ CFU/mL). Incubate at 37 °C for 18 hours, then measure the OD600 value of each well using an ELISA reader to determine bacterial growth.

### 2.8 Biofilm formation and disruption assay

Biofilm Formation Inhibition Assay: A 1×10^6^ CFU/mL suspension of hv*Kp* was added to a 96-well plate along with niacin at varying concentrations. The plates were incubated at 37 °C for 24 hours. The culture supernatant was discarded, and the wells were gently washed three times with sterile PBS. Fixed with methanol for 15 min, stained with 0.1% crystal violet for 10 min, washed, air-dried, and examined under a microscope to observe biofilm morphology. Add 33% glacial acetic acid to dissolve the crystal violet, and measure the optical density (OD) at 590 nm using a microplate reader.

Mature biofilm disruption assay: Incubate hv*Kp* bacterial suspension at 37 °C for 24 h to form a mature biofilm. Discard the bacterial suspension, add niacin at gradient concentrations, and continue incubation for 24 h. Subsequent staining, quantification, and microscopic observation steps are the same as above.

### 2.9 Animal models

SPF-grade male C57BL/6 mice, 6–8 weeks old, weighing 18–22 g, were purchased from Jicui Yaokang Biotechnology Co., Ltd (Jiangsu, China). All supplements including food, water, and other nutrients were autoclaved, and animals were kept in the animal facilities. All surgeries were performed under anesthesia with Tribromoethanol, and utmost efforts were taken to minimize suffering.

Mice were anesthetized by inhalation of 5% isoflurane (RWD, R510-22-10) via an animal anesthesia machine (RWD, R640), and anesthesia was maintained with a 2% concentration. Secure the mouse’s head in a stereotaxic apparatus and adjust the head to a horizontal position, ensuring the skull is symmetrical on both sides. Make an incision of approximately 1 cm along the midline of the sagittal suture and dissect the periosteum to fully expose the anterior fontanelle and the sagittal suture. Using the anterior fontanelle as the coordinate origin, mark the right lateral ventricle puncture site: AP (anterior-posterior axis): -0.5 to -0.6 mm (posterior to the anterior fontanelle); ML (medial-lateral axis): +1.2 to +1.3 mm (to the right of the midline); DV (dorsoventral axis): -1.8 to -2.0 mm (toward the cranial surface). Use a dental drill to penetrate the skull at the marked points, preserving the integrity of the dura mater and avoiding damage to brain tissue. Using a 32-gauge needle, inject 10 μL of a bacterial suspension (1 × 10^6^ CFU/mL) into the ventricle at a rate of 0.5 μL/min. Leave the needle in place for 5 minutes after injection to allow the bacterial solution to diffuse fully and prevent backflow. Remove the needle slowly and at a constant speed. Apply gentle pressure to the puncture site with a sterile cotton ball to stop bleeding. Close the scalp with absorbable sutures and disinfect the wound with povidone-iodine (Mook-Kanamori et al., 2012).

The mice were divided into five groups: WT, hv*Kp* (1 × 10^4^ CFU), hv*Kp* + niacin (1.8 mg/g, i.p.), pre-niacin (1.8 mg/g, 6h, i.p.) + hv*Kp*, and pre-niacin + hv*Kp* + GM (10 mg/kg, i.p.). Following infection, the mice’s survival status and time of death were continuously monitored and recorded. Kaplan-Meier survival curves were plotted to analyze the survival prognosis of mice in each group.

### 2.10 Bacterial load assay

Twenty-four hours after infection, the mice were anesthetized with sodium pentobarbital (50–90 mg/kg, i.p.) and euthanized. Brain tissue, cerebrospinal fluid, blood, liver, and kidney tissues were collected under sterile conditions. After weighing, the samples were homogenized in sterile PBS, serially diluted, and spread onto MHA solid medium. The plates were incubated at 37 °C in an inverted incubator for 18 hours. Colonies were counted, and the bacterial load per gram of tissue (CFU/g) was calculated.

### 2.11 Blood-brain barrier permeability assay (Evan’s Blue leakage test)

Inject 2% Evan’s Blue solution (5 mL/kg) into the tail vein of mice; after 1 hour of circulation, the mice were anesthetized with sodium pentobarbital (50–90 mg/kg, i.p.) and euthanized. Perfuse the heart with saline until the effluent is colorless, then weigh the brain tissue. After immersion in formamide, incubate at 37 °C for 48 hours to extract the Evans Blue from the tissue. Measure the absorbance at 620 nm using a microplate reader, and calculate the Evans Blue content in the brain tissue based on a standard curve to reflect blood-brain barrier permeability.

### 2.12 Statistical analysis

Data were obtained from at least three independent experiments. Data are presented as mean ± standard error of the mean (SEM). An unpaired t-test was used for comparisons between two groups, while one-way or two-way analysis of variance (ANOVA) was used for analyses involving multiple groups. One-way ANOVA and unpaired t-tests were performed using GraphPad Prism 8 software; a P-value of <0.05 was considered statistically significant.

## 3 Results

### 3.1 hv*Kp* infection induces apoptosis in HCMEC

**Figure 1:**
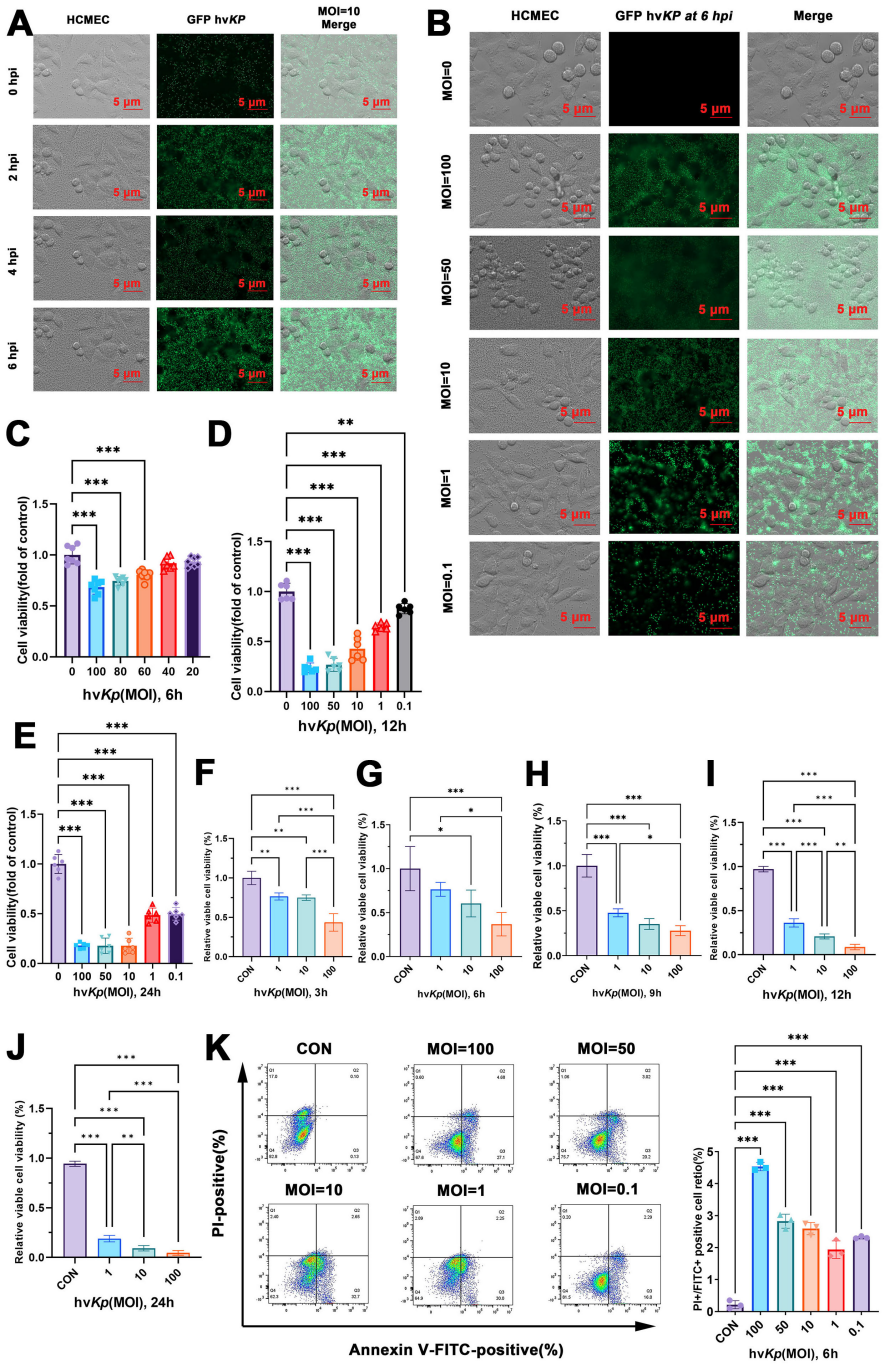
GFP**-**hv*Kp* invades HCMEC in a time- and dose-dependent manner and induces apoptosis. **(A)** Live-cell imaging demonstrates the dynamic process of GFP-hv*Kp* invasion of HCMEC at an MOI of 10. The rows show the optical morphology of HCMEC, GFP-hv*Kp*, and a merged view, while the columns correspond to infection time points of 0, 2, 4, and 6 hours; **(B)** Live-cell imaging demonstrates the invasion of HCMEC by GFP-hv*Kp* at different MOIs 6 hours post-infection. The rows show the optical morphology of HCMEC, GFP-hv*Kp*, and a merged view; the columns correspond to MOIs of 0, 0.1, 1, 10, 50, and 100, respectively. **(C–E)** Cell viability was assessed using the CCK-8 assay following infection of HCMEC with GFP-labeled hv*Kp* at varying MOIs for 6 h **(C)**, 12 h **(D)**, and 24 h **(E)**; **(F–J)** Trypan blue staining to determine the relative viability of HCMEC infected with hv*Kp* at varying MOIs after 3 h **(F)**, 6 h **(G)**, 9 h **(H)**, 12 h **(I)**, and 24 h **(J)**; **(K)** Annexin V-FITC/PI double-staining flow cytometry analysis of apoptosis in HCMEC 6 h after infection with hv*Kp* at varying MOI gradients; All data are expressed as mean ± SEM; one-way ANOVA with Tukey’s posttest;*P < 0.05, **P < 0.01, ***P < 0.001.

Using a GFP-labeled hv*Kp* strain, we established both a time-dependent infection model with an multiplicity of infection (MOI) of 10 and a dose-dependent infection model with a 6-hour incubation period. The invasion dynamics of hv*Kp* in HCMEC were observed using a live-cell imaging system. The results showed that during MOI = 10 infection, intracellular hv*Kp* fluorescence signals increased significantly over time within 4 hours, accompanied by rapid bacterial proliferation; by 6 h, hv*Kp* proliferation slowed, and some HCMEC exhibited signs of damage such as cell membrane invagination (**Figure 1A**). This finding suggests that in the early stages of hv*Kp* infection, prior to reaching the proliferation plateau, intracellular proliferation is the central process, subsequently leading to acute damage in host cells resembling apoptosis (Kerr et al., 1972). When cells were fixed at 6 h post-infection, the intracellular hv*Kp* fluorescence signal increased in a dose-dependent manner as the MOI rose from 0.1 to 100; clear invasion was observed even at an MOI as low as 0.1, and intracellular bacterial load reached a high level at MOI ≥ 10 (**Figure 1B**).

We used the CCK-8 assay to assess HCMEC viability 6, 12, and 24 hours after infection with hv*Kp* at different initial MOIs. The results showed that at 6 hours post-infection, only hv*Kp* infection with an MOI ≥ 60 significantly reduced cell viability (**Figure 1C**); At 12 hours post-infection, hv*Kp* infection with an MOI ≥ 0.1 significantly suppressed cell viability (**Figure 1D**); And at 24 hours post-infection, HCMEC viability in the groups with an MOI ≥ 10 dropped to extremely low levels (**Figure 1E**). The absorbance value measured by the CCK-8 assay depends on mitochondrial dehydrogenase activity (Elmore, 2007); To further confirm whether the decrease in CCK-8 signal reflects actual cell death, we used trypan blue staining to directly assess cell membrane integrity.

To further validate the actual survival status of HCMEC under hv*Kp* infection, we performed trypan blue staining. The results showed that the lethal effect of hv*Kp* on HCMEC was time- and dose-dependent, fully consistent with the dynamics of intracellular bacterial proliferation (**Figures 1F–J**). Flow cytometry results using Annexin V-FITC/PI showed that the control group had extremely low levels of apoptosis, with 0.13% of cells in the early stage of apoptosis and 0.10% in the late stage; As the MOI increased from 0.1 to 100, the proportion of cells in the early stage of apoptosis rose from 16.0% to 27.1%, and the proportion of cells in the late stage of apoptosis rose from 2.29% to 4.68%, with the proportion of cells in the late stage of apoptosis peaking (4.68%) in the MOI = 100 group (**Figure 1K**). These results indicate that hv*Kp* induces apoptosis in HCMEC in a dose-dependent manner; even at the lowest MOI (0.1) after 6 hours of infection, it significantly increased the proportion of apoptotic cells. This suggests that apoptosis is the primary mechanism by which hv*Kp* induces damage in HCMEC.

### 3.2 hv*Kp* induces apoptosis in HCMEC via ROS accumulation

**Figure 2:**
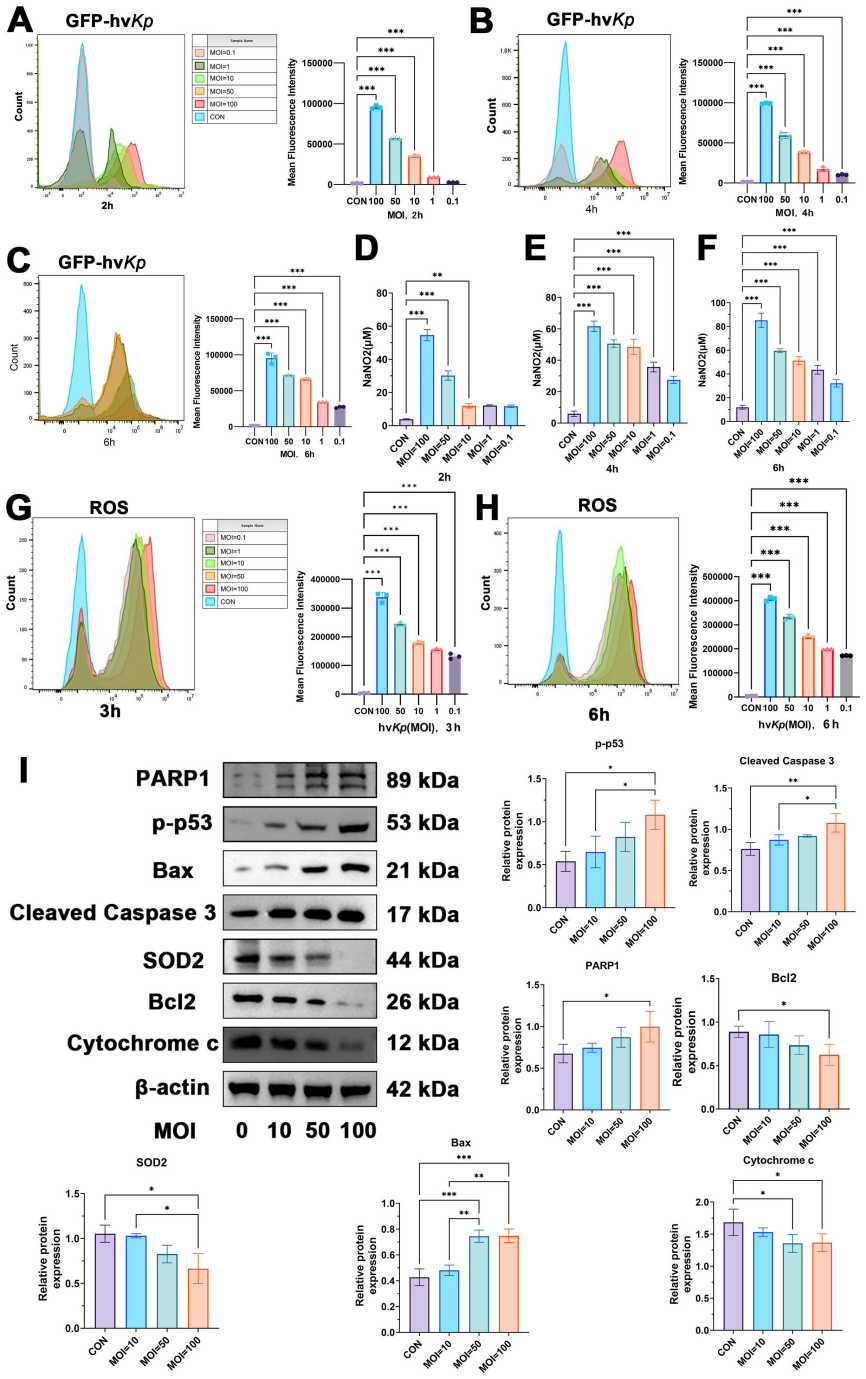
The role of hv*Kp* in inducing mitochondrial oxidative stress in HCMEC. **(A–C)** Flow cytometry analysis of intracellular bacterial load in GFP-labeled hv*Kp* at varying MOIs (0, 0.1, 1, 10, 50, 100) after infecting HCMEC for 2 h **(A)**, 4 h **(B)**, and 6 h **(C)**, including representative flow cytometry histograms and quantitative analysis of the mean intracellular GFP fluorescence intensity; **(D–F)** NaNO_2_ concentrations in cell culture supernatants 2 h **(D)**, 4 h **(E)**, and 6 h **(F)** after infection of HCMEC with hv*Kp* at varying MOIs; **(G, H)** Flow cytometric analysis of mitochondrial ROS levels in HCMEC 3 h **(G)** and 6 h **(H)** after infection with hv*Kp* at varying MOIs. **(I)** Western blot analysis of apoptosis pathway and antioxidant-related protein expression in HCMEC infected with hv*Kp* at different MOIs for 6 hours. All data are expressed as mean ± SEM (n=3); one-way ANOVA with Tukey’s posttest;*P < 0.05, **P < 0.01, ***P < 0.001.

To establish a stable *in vitro* model for hv*Kp* infection of HCMEC and to determine the intracellular invasion efficiency of hv*Kp* under different infection conditions, we infected HCMEC with a GFP-tagged hv*Kp* at varying MOIs. Cells were harvested 2, 4, and 6 hours post-infection, and the mean fluorescence intensity (MFI) of intracellular GFP fluorescence was measured by flow cytometry to quantitatively analyze the intracellular hv*Kp* load. The results showed that intracellular GFP fluorescence intensity increased in a gradient with rising hv*Kp* MOI, with the MOI = 100 group reaching peak fluorescence intensity at all time points (**Figures 2A–C**). These findings confirm that hv*Kp* infection at MOI = 100 for 6 h can establish a stable *in vitro* infection model.

Oxidative stress constitutes a key mechanism of hv*Kp* infection, inducing a cascade of inflammatory responses and cellular damage (Cui et al., 2025). Nitric oxide (NO) is a key effector molecule that mediates cellular inflammatory activation and functional impairment following bacterial infection (Bogdan, 2001). However, due to its short half-life, it readily oxidizes in aqueous solutions to form NO_2_^−^ and NO_3_^−^. Therefore, we used the Griess assay to measure the concentration of the stable extracellular metabolite NO_2_^−^, which indirectly reflects the levels of NO production and release (Granger et al., 1996). The results showed that hv*Kp* infection significantly promoted NO release from HCMEC. At 2 h post-infection, only high-dose hv*Kp* infection (MOI ≥ 50) significantly increased the concentration of NaNO_2_ in the cell culture supernatant. By 6 h post-infection, even infection at an MOI as low as 0.1 significantly induced NO release from HCMEC (**Figures 2D–F**).

Excessive production of mitochondrial ROS is a key upstream event in cellular oxidative stress damage and the initiation of apoptosis. We used flow cytometry to examine the regulatory effects of hv*Kp* infection on mitochondrial oxidative stress levels in HCMEC. The results showed that hv*Kp* infection significantly induced excessive ROS production in HCMEC in a dose-dependent manner; at 3 h post-infection, hv*Kp* infection with an MOI ≥ 0.1 significantly increased the mean fluorescence intensity of intracellular ROS; by 6 h post-infection, ROS levels in all MOI groups had further increased, suggesting that hv*Kp* infection continuously exacerbates mitochondrial oxidative stress damage in HCMEC (**Figures 2G, H**). P53, as a key oxidative stress-sensing protein, is activated under ROS stress, leading to the transcriptional upregulation of the pro-apoptotic protein Bax and the inhibition of the anti-apoptotic protein Bcl2, thereby resulting in increased mitochondrial outer membrane permeability (Saha and Kundu, 2021). Western blot results indicate that, 6 hours post-infection, hv*Kp* infection upregulates the expression of pro-apoptotic proteins PARP1, p-p53, Bax, and cleaved caspase-3, while simultaneously downregulating the levels of the anti-apoptotic protein Bcl2, the antioxidant protein SOD2, and cytosolic cytochrome c (**Figure 2I**). This suggests that hv*Kp* infection activates the mitochondrial apoptosis pathway in HCMEC by compromising the cellular antioxidant defense system.

### 3.3 The inhibitory effect of niacin on hv*Kp* proliferation and biofilm formation

As an essential water-soluble vitamin for human, niacin has been shown to possess multiple biological activities, including anti-inflammatory, antioxidant, and modulatory effects on bacterial virulence. However, its antibacterial and endothelial protective roles in hv*Kp* infection remain unclear. To elucidate the modulatory effects of niacin on the intrinsic virulence of hv*Kp*, we systematically investigated the impact of niacin on hv*Kp* proliferation and biofilm formation, and evaluated its synergistic antibacterial activity with commonly used clinical antibiotics.

**Figure 3:**
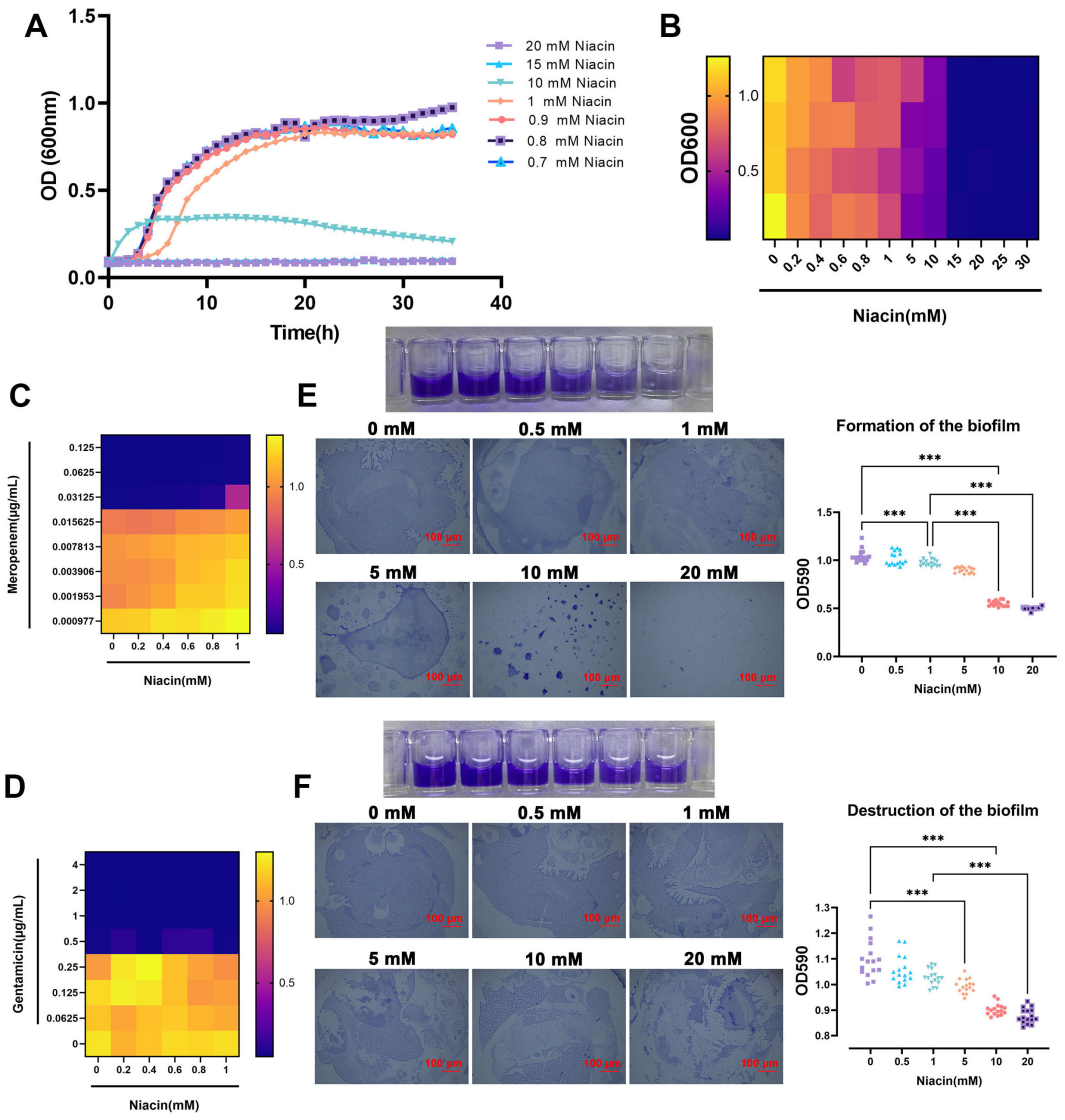
The inhibitory effects of niacin on hv*Kp* proliferation and biofilm formation. **(A)** Growth curve of hv*Kp* after treatment with niacin at gradient concentrations, showing the dynamic changes in the bacterial culture at 600 nm (OD600) over 40 hours of continuous measurement; **(B)** Antimicrobial assay to determine the minimum inhibitory concentration (MIC) of niacin against hv*Kp*; **(C, D)** Checkerboard microdilution assay to evaluate the synergistic antimicrobial effects of niacin in combination with meropenem **(C)** and GM **(D)** against hv*Kp*; **(E, F)** Crystal violet staining to assess the effects of gradient concentrations of niacin on hv*Kp* biofilm formation **(E)** and disruption of mature biofilms **(F)**, including representative microscopic images, stained specimens, and quantitative analysis results at 590 nm (OD 590); all data are expressed as mean ± SEM; one-way ANOVA with Tukey’s posttest;*P < 0.05, **P < 0.01, ***P < 0.001.

Growth curve assays demonstrated that niacin concentration-dependently inhibited the *in vitro* proliferation of hv*Kp*. Concentrations of niacin ≥ 1 mM significantly slowed the growth rate of hv*Kp*, and 15 mM niacin completely inhibited hv*Kp* proliferation (**Figure 3A**). The results of the antibacterial assay showed that the minimum inhibitory concentration (MIC) of niacin against hv*Kp* was 15 mM (**Figure 3B**). The results of checkerboard microdilution assay confirmed that the combination of niacin at concentrations ranging from 0 to 1 mM with meropenem or GM did not enhance the antibacterial activity of these two drugs (FICI<4) (Doern, 2014), indicating that niacin concentrations of 1 mM or less do not enhance the antimicrobial activity of meropenem or GM. (**Figures 3C, D**). Crystal violet staining and microscopic observations demonstrated that niacin concentration-dependently inhibited hv*Kp* biofilm formation and significantly disrupted established mature biofilms. At a concentration of 1 mM, niacin inhibits the formation of a fully developed, network-like biofilm by hv*Kp* (*P* < 0.001). As the niacin concentration increases to 5 mM, the hv*Kp* biofilm gradually breaks down from a continuous network structure into smaller, discrete network structures (*P* < 0.001) (**Figures 3E, F**).

### 3.4 Niacin can attenuate hv*Kp*-induced apoptosis in HCMEC

**Figure 4:**
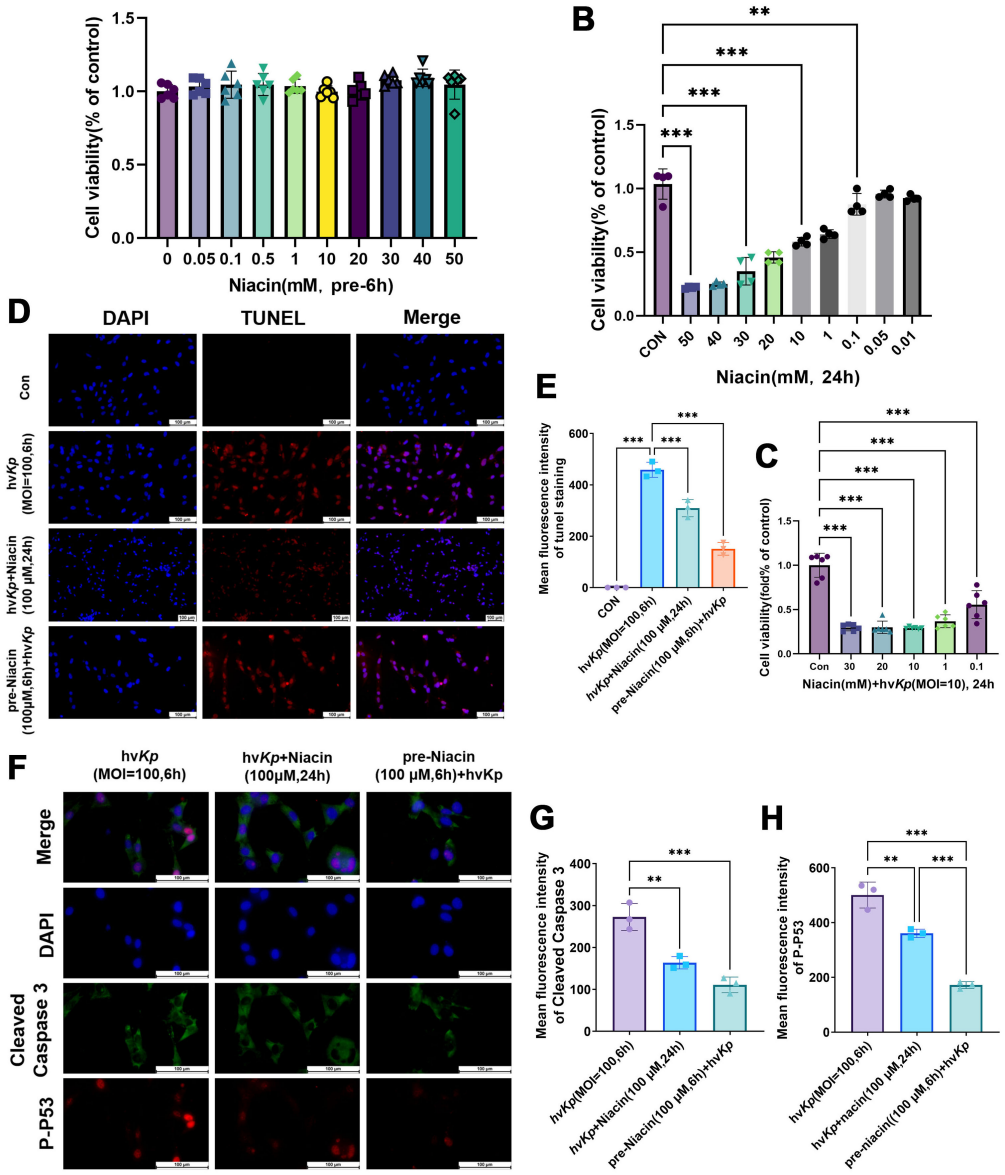
Niacin alleviates hv*Kp*-induced apoptotic damage. **(A, B)** The CCK-8 assay was used to assess the effects of niacin pretreatment at gradient concentrations for 6 h **(A)** and continuous treatment for 24 h **(B)** on HCMEC viability, evaluating the cytotoxicity of niacin. **(C)** The CCK-8 assay was used to assess the reversal of HCMEC viability decline caused by hv*Kp* infection (MOI = 10) for 24 h following treatment with gradient concentrations of niacin. **(D, E)** TUNEL staining was used to assess the effect of niacin intervention on HCMEC apoptosis induced by hv*Kp* infection. The columns show four groups of cells from top to bottom: CON, hv*Kp* (MOI = 100, 6h), hv*Kp* + niacin (100 μM, 24h), and pre-niacin (100 μM, 6h) + hv*Kp* **(D)** Bar chart showing quantitative analysis of TUNEL-positive signals **(E)**. **(F–H)** Immunofluorescence staining was used to detect the expression of cleaved caspase-3 and p-p53 in HCMEC from each group. The row shows fluorescence images of cells treated with hv*Kp* (MOI = 100, 6 h), hv*Kp* + niacin (100 μM, 24 h), and pre-niacin(100 μM, 6 h) + hv*Kp*, while The columns correspond to p-p53, cleaved caspase-3, DAPI, and composite images **(F)**; Bar chart showing quantitative analysis of cleaved caspase-3 **(G)**; Bar chart showing quantitative analysis of p-p53 **(H)**. All data are expressed as mean ± SEM; one-way ANOVA with Tukey’s posttest;*P < 0.05, **P < 0.01, ***P < 0.001.

The results of cell proliferation and toxicity assays indicate that, when pretreated for 6 hours, niacin concentrations of ≤50 mM had no significant effect on the baseline survival rate of HCMEC (**Figure 4A**). Treatment with niacin concentrations of ≤ 0.1 mM for 24 hours did not exhibit significant cytotoxicity toward HCMEC, confirming that niacin is non-cytotoxic to HCMEC when administered for short periods across a wide range of concentrations (**Figure 4B**). Concurrently, niacin significantly reversed the decline in HCMEC viability induced by hv*Kp* infection at an MOI of 10, demonstrating a clear endothelial protective effect within the safe concentration range (**Figure 4C**).

To further investigate the protective effect of niacin against hv*Kp*-induced HCMEC apoptosis, we first used TUNEL staining to detect the impact of niacin intervention on HCMEC apoptosis and DNA damage levels following hv*Kp* infection. TUNEL staining specifically marks DNA strand breaks occurring during the apoptosis process; the proportion of positive cells directly reflects the extent of apoptosis and correlates with the activation level of PARP1, a key protein in DNA damage repair. TUNEL staining results showed that hv*Kp* infection significantly increased the intensity of TUNEL-positive fluorescence signals in HCMEC, significantly inducing DNA damage and apoptosis in endothelial cells; whereas niacin treatment (hv*Kp* + niacin) and pretreatment (pre-niacin + hv*Kp*) both significantly reduced the intensity of TUNEL-positive signals, decreased the number of apoptotic cells, and effectively inhibited hv*Kp*-induced endothelial cell apoptosis (**Figures 4D, E**). Immunofluorescence results further confirmed that following hv*Kp* infection, the fluorescence intensity of cleaved caspase-3 and p-p53 in HCMEC was significantly elevated, whereas niacin pretreatment significantly reduced the fluorescence signal intensity of both, reaffirming that niacin effectively inhibits the activation of hv*Kp*-induced apoptotic pathways and exerts an endothelial protective effect (**Figures 4F–H**).

### 3.5 Niacin alleviates hv*Kp*-induced apoptosis in HCMEC by reducing ROS levels

**Figure 5:**
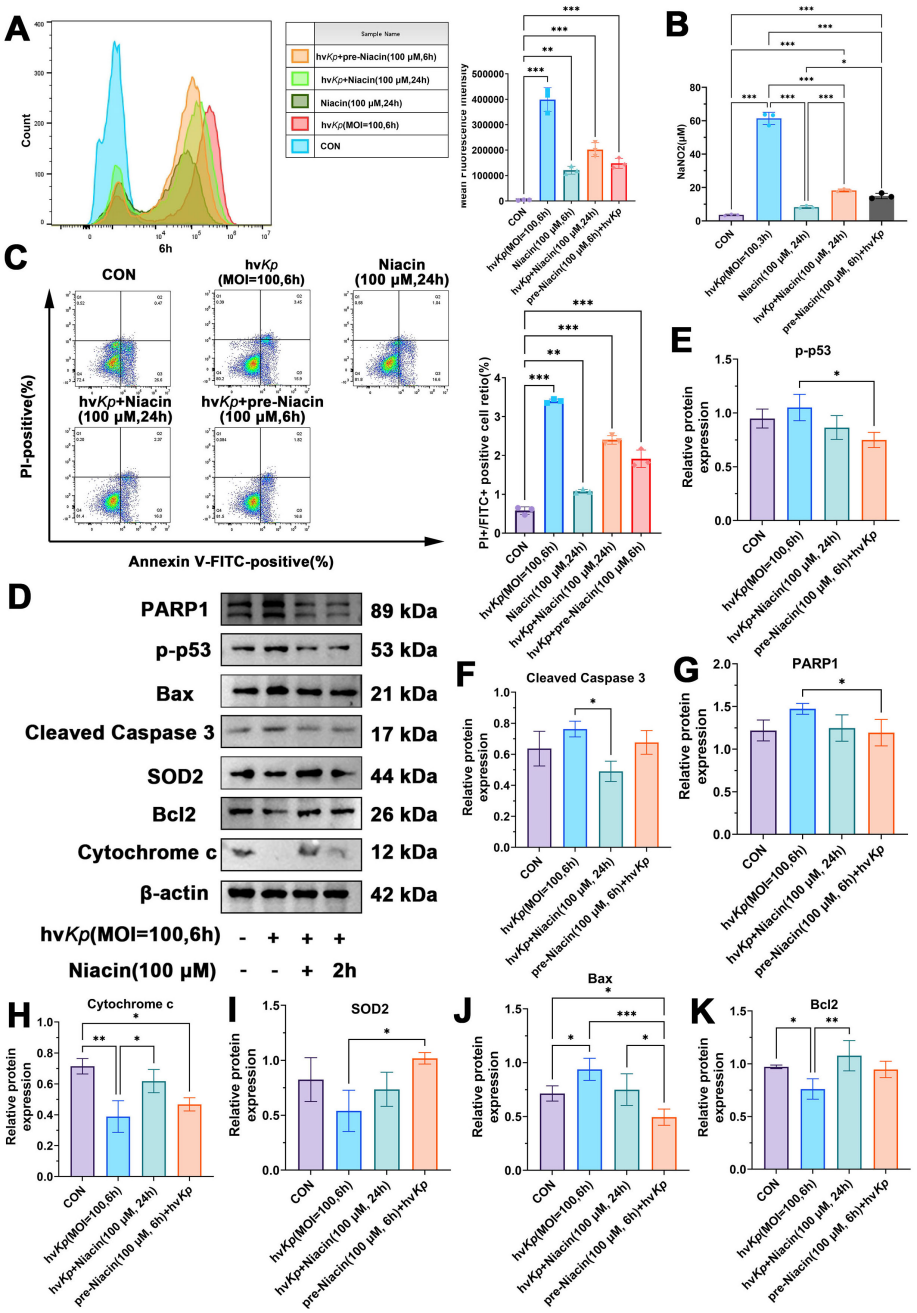
Niacin inhibits the activation of the HCMEC apoptosis pathway by reducing mitochondrial oxidative stress. **(A)** Quantitative analysis of intracellular ROS levels and mean fluorescence intensity in HCMEC from each group by flow cytometry. **(B)** NaNO_2_ concentrations in cell culture supernatants from different treatment groups. **(C)** Apoptosis rates in HCMEC from each group as determined by Annexin V-FITC/PI double-staining flow cytometry. **(D–K)** Western blot analysis of apoptosis pathway and antioxidant-related protein expression in each group. All data are expressed as mean ± SEM; one-way ANOVA with Tukey’s posttest;*P < 0.05, **P < 0.01, ***P < 0.001.

To elucidate the protective effects and regulatory role of niacin against oxidative stress and apoptosis-induced damage in HCMEC following hv*Kp* infection, we first used flow cytometry to assess the impact of niacin intervention on intracellular ROS production after hv*Kp* infection. The results showed that hv*Kp* infection (MOI = 100, 6 h) significantly increased the mean fluorescence intensity of intracellular ROS in HCMEC, whereas co-treatment with niacin (hv*Kp* + niacin, 100 μM) and pretreatment with niacin (pre-niacin + hv*Kp*, 100 μM) both significantly reversed this effect, with the inhibitory effect of niacin pretreatment being more pronounced (**Figure 5A**). Concurrent detection of the NO metabolite NaNO_2_ in cell culture supernatants showed that niacin intervention significantly reduced the hv*Kp*-induced abnormal release of NO, thereby alleviating infection-mediated inflammation and oxidative stress damage (**Figure 5B**).

Based on this, we performed quantitative analysis of the apoptosis rate using Annexin V-FITC/PI double-staining flow cytometry. The results showed that the proportion of late apoptotic cells in the group infected with hv*Kp* alone (MOI = 100, 6 h) increased significantly to 3.45% compared with the control group, while the proportion of early apoptotic cells decreased to 15.9%. Compared with the hv*Kp*-infected group, the proportion of late apoptotic cells in the group infected with concurrent niacin (hv*Kp* + niacin, 100 μM) and the group pretreated with niacin prior to infection (hv*Kp* + pre-niacin, 100 μM) decreased to 2.37% and 1.82%, respectively (**Figure 5C**). These results suggest that niacin can significantly reduce hv*Kp*-induced apoptotic damage, with pre-treatment yielding superior effects. To further elucidate the core molecular mechanism by which niacin inhibits apoptosis, we performed western blot analysis to determine whether the expression of key proteins in the apoptotic pathway was reduced following niacin treatment. The results showed that niacin intervention significantly reversed the abnormal expression of apoptosis-related proteins induced by hv*Kp* infection: downregulating PARP1, p-p53, cleaved caspase-3, and the pro-apoptotic protein Bax, while simultaneously restoring the expression levels of the anti-apoptotic protein Bcl2, the endogenous antioxidant protein SOD2, and cytosolic Cytochrome c, thereby blocking the excessive activation of the mitochondrial apoptotic pathway and exerting a protective effect (**Figures 5D–K**).

### 3.6 Niacin significantly improves survival outcomes and reduces tissue damage in mice with hv*Kp* meningitis

**Figure 6:**
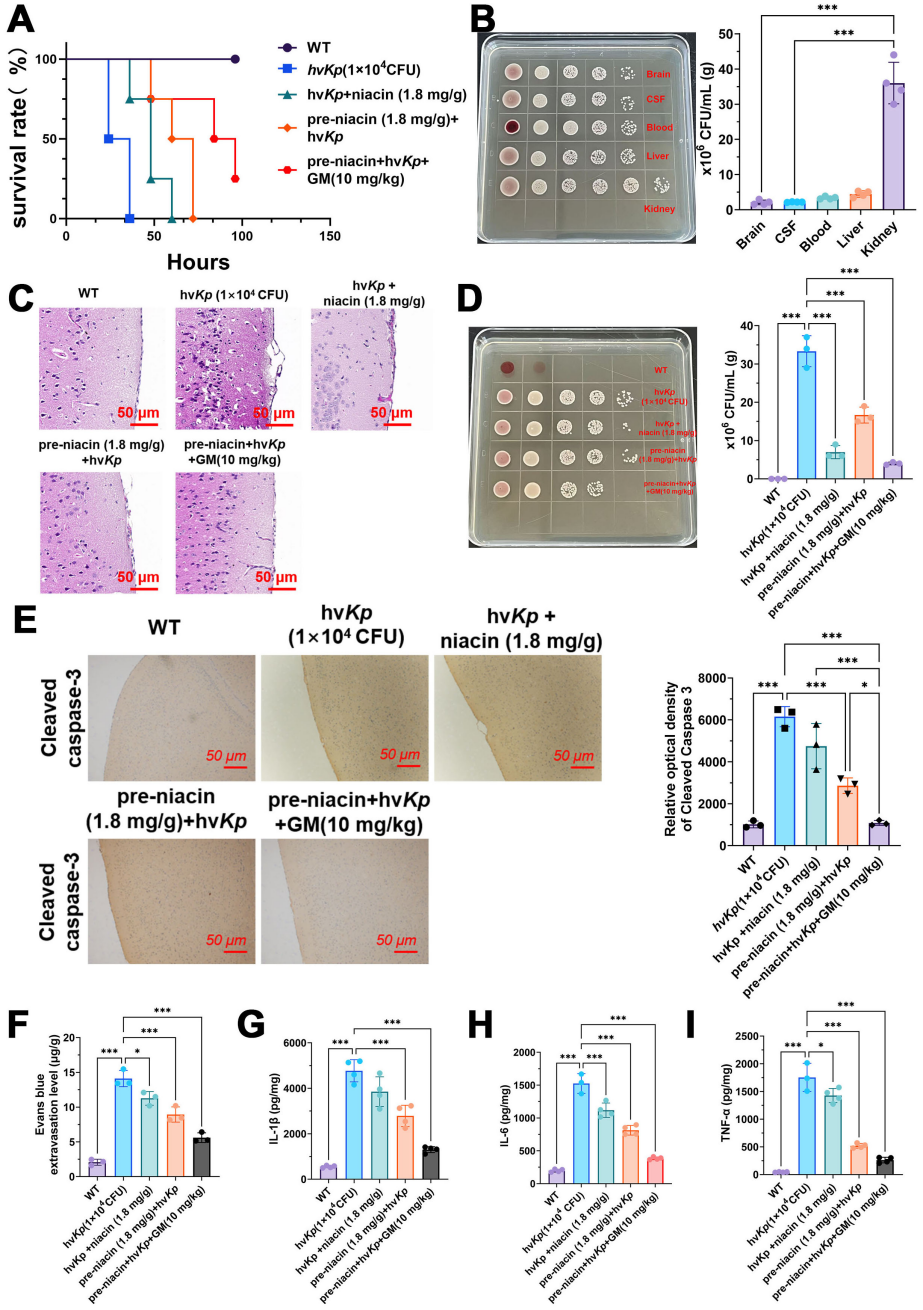
*In vivo* protective effects of niacin against the hv*Kp*-Induced meningitis model in mice. **(A)** Survival rates of mice in each group following hv*Kp*-induced meningitis. **(B)** hv*Kp* load in various tissues and organs of mice with meningitis, as determined by plate count. **(C)** Pathological damage in brain tissue of mice in each group, as assessed by HE staining. **(D)** Bacterial load in brain tissue of mice in each group, as determined by plate count. **(E)** Expression of cleaved caspase-3 in brain tissue of mice in each group, as assessed by immunohistochemical staining, with quantitative analysis of mean optical density. **(F)** Blood-brain barrier permeability in mice of each group, as assessed by the Evans blue leakage assay. **(G–I)** ELISA analysis of the expression levels of pro-inflammatory cytokines IL-1β **(G)**, IL-6 **(H)**, and TNF-α **(I)** in mouse brain tissue from each group. All data are expressed as mean ± SEM; one-way ANOVA with Tukey’s posttest;*P < 0.05, **P < 0.01, ***P < 0.001.

To verify the protective effect of niacin against central nervous system infection by hv*Kp in vivo*, we established a mouse model of hv*Kp* meningitis. Survival analysis results showed that hv*Kp* infection led to rapid death in mice, with the survival rate dropping to 0 within 72 hours of infection; In contrast, niacin treatment prolonged survival. Mice in the hv*Kp* + niacin group maintained approximately 75% survival at 36 h, although all mice died by 60 h. Pre-treatment with niacin further delayed mortality, with approximately 50% survival maintained until 60 h. Notably, the combined intervention of pre-niacin + hv*Kp* + GM showed the strongest protective effect, with approximately 50% survival at 84 h and 25% survival remaining at 96 h (**Figure 6A**).

Results of tissue bacterial load assays revealed high levels of hv*Kp* colonization in the brain tissue, cerebrospinal fluid, blood, and liver and kidney tissues of mice following hv*Kp* infection, suggesting systemic dissemination of the infection; niacin intervention significantly reduced hv*Kp* load in all tissues and organs, particularly by markedly inhibiting hv*Kp* colonization in the central nervous system; the combination of niacin pretreatment and GM exerted the optimal antibacterial protective effect (**Figures 6B, D**).

Histopathological examination further confirmed the protective effect of niacin. H&E staining showed that hv*Kp* infection caused obvious meningeal thickening, inflammatory cell infiltration, and tissue structural disruption. Niacin intervention significantly alleviated hv*Kp*-induced pathological damage in brain tissue and reduced inflammatory cell infiltration. The pre-niacin + hv*Kp* + GM group exhibited the mildest pathological injury, approaching the normal appearance observed in the control group (**Figure 6C**). Compared with the WT group, which showed a low Evans blue level of approximately 2 μg/g, hv*Kp* infection significantly increased Evans blue leakage to approximately 14 μg/g, indicating severe BBB disruption. Niacin treatment reduced Evans blue extravasation to approximately 11 μg/g, while pre-treatment with niacin further decreased it to approximately 9 μg/g. The pre-niacin + hv*Kp* + GM group showed the strongest protective effect, reducing Evans blue leakage to approximately 5–6 μg/g. These data suggest that niacin helps preserve BBB integrity during hv*Kp* infection (**Figure 6F**).

Inflammatory factor detection results showed that hv*Kp* infection significantly upregulated the expression of pro-inflammatory factors IL-1β, IL-6, and TNF-α in mouse brain tissue, inducing a central inflammatory cascade. Niacin intervention significantly downregulated the abnormally elevated expression of these pro-inflammatory factors, thereby suppressing the central inflammatory activation induced by hv*Kp* infection (**Figures 6G–I**). Immunohistochemical staining showed that hv*Kp* infection markedly increased cleaved caspase-3 expression in brain tissue. Quantitative analysis demonstrated that the relative optical density of cleaved caspase-3 increased from approximately 1,000 in the WT group to approximately 6,200 in the hv*Kp* group. Niacin treatment reduced this value to approximately 4,700, while pre-treatment with niacin further reduced it to approximately 2,800. The pre-niacin + hv*Kp* + GM group showed the most pronounced reduction, with cleaved caspase-3 expression decreasing to nearly baseline levels. These results indicate that niacin attenuates hv*Kp*-induced apoptosis in brain tissue (**Figure 6E**).

## 4 Discussion

Central nervous system infections caused by hv*Kp* (such as meningitis and brain abscesses) are characterized by high mortality rates and poor prognoses; the key pathogenic mechanism lies in the breach of the blood-brain barrier (Carrie et al., 2019). However, the mechanisms by which hv*Kp* damages brain microvascular endothelial cells and compromises BBB integrity remain incompletely understood. In the present study, we showed that hv*Kp* induces progressive injury in HCMEC, accompanied by intracellular bacterial accumulation, excessive ROS generation, and activation of the p53-mediated mitochondrial apoptotic pathway. Importantly, niacin attenuated hv*Kp*-induced endothelial damage by reducing ROS accumulation and apoptosis, suggesting that modulation of host oxidative stress may represent a potential adjunctive strategy for hv*Kp*-associated central nervous system infection.

A notable finding of this study is that hv*Kp* infection of HCMEC exhibits a two-step pattern characterized by “intracellular proliferation followed by apoptosis induction.” This finding differs from the traditional pathogenic infection pattern of “immediate damage initiation upon adhesion” (Cui et al., 2025), suggesting that hv*Kp* may breach the blood-brain barrier defenses through a strategy of “bacterial accumulation and amplification of virulence effects.” Further confirmation via CCK-8, trypan blue staining, and apoptosis assays demonstrated that hv*Kp*-induced damage to HCMEC is both MOI- and time-dependent. This characteristic may be related to the expression pattern of hv*Kp* virulence factors—once the bacterial population reaches a certain density, the quorum sensing system activates the release of virulence factors (such as capsular polysaccharides and iron carriers) (Russo and Marr, 2019), thereby initiating the damage program against HCMEC. Furthermore, the observation of intracellular bacterial accumulation indicates that hv*Kp* may persist or replicate within endothelial cells before obvious cell death occurs. This is biologically important because intracellular localization may allow bacteria to evade extracellular immune defenses and antibiotic exposure, while simultaneously inducing host cell stress responses. In the context of the BBB, such intracellular persistence may weaken endothelial integrity and facilitate further bacterial penetration into the central nervous system. Therefore, hv*Kp*-mediated endothelial injury may represent a critical early event in the pathogenesis of hv*Kp* meningitis.

At the mechanistic level, this study confirms that oxidative stress is a key upstream regulatory event in hv*Kp*-induced apoptosis of HCMEC. hv*Kp* infection induces a sharp increase in intracellular ROS and NO levels. NO can rapidly react with ROS to form nitrous peroxide (ONOO^−^), which possesses strong oxidizing and nitrating capabilities and can lead to protein nitrosylation, lipid peroxidation, mitochondrial damage, and DNA damage (Beckman et al., 1990). Brain endothelial cells are particularly vulnerable to oxidative injury because mitochondrial dysfunction and cytoskeletal instability can disrupt tight junction organization and increase vascular permeability. Our results are consistent with previous studies showing that oxidative stress contributes to BBB breakdown and neurological injury in bacterial meningitis and other neuroinflammatory diseases (Mittal et al., 2014). In this study, the increase in ROS following hv*Kp* infection suggests that oxidative stress is not simply a secondary consequence of cell injury, but may serve as an upstream mediator that drives mitochondrial dysfunction and apoptosis.

P53 is a key stress-responsive transcription factor that integrates signals from oxidative stress, DNA damage, and mitochondrial injury (Vousden and Prives, 2009). Under severe stress conditions, p53 can regulate members of the Bcl2 family, promote mitochondrial outer membrane permeabilization, enhance cytochrome c release, and activate caspase-dependent apoptosis (Saha and Kundu, 2021). In our model, hv*Kp*-induced ROS accumulation was associated with activation of p53-related mitochondrial apoptotic signaling, suggesting that oxidative stress shifts the balance between survival and death signals toward apoptosis. The flow cytometry results further support this interpretation. The increase in both early and late apoptotic cells indicates that hv*Kp* not only initiates apoptosis but also promotes progression toward irreversible membrane damage and terminal cell death. This distribution is meaningful because early apoptosis may represent a potentially reversible stage, whereas late apoptosis reflects more advanced endothelial injury that may directly compromise barrier continuity. The ability of niacin to reduce apoptotic cell populations suggests that it may interrupt this injury process and preserve endothelial viability.

As an important precursor of NAD^+^, niacin is known to cross the blood-brain barrier and participate in the regulation of cellular energy metabolism and redox homeostasis (Wuerch et al., 2023). Based on these mechanisms, we investigated the protective potential of niacin. A key finding of this study was the identification of strict dose-dependence and time-specificity in the effects of niacin. We found that the protective effects of niacin exhibit strict dose- and time-specificity: while high concentrations of niacin can directly inhibit hv*Kp* proliferation, they are accompanied by host cell toxicity. At a physiological concentration of 100 μM, however, niacin exhibits no direct antibacterial activity but can alleviate HCMEC apoptosis by reversing hv*Kp*-induced oxidative stress damage. This suggests that the protective effect of niacin primarily stems from its antioxidant and anti-apoptotic host cell regulatory functions, rather than direct antibacterial activity. Additionally, the protective effect of niacin pretreatment was superior to post-infection administration, providing guidance for clinical treatment strategies: for high-risk populations with compromised immunity (such as diabetic patients), prophylactic supplementation with an appropriate dose of niacin prior to infection may prevent hv*Kp*-associated CNS infections, or niacin may be administered in combination with treatment during the early stages of infection to mitigate blood-brain barrier damage. Building on this, the hv*Kp* mouse meningitis model established in this study further validated the protective effects of niacin in a complete *in vivo* physiological and pathological environment. *In vivo* experimental results confirmed that niacin intervention effectively maintains blood-brain barrier integrity, inhibits central inflammatory cascades, and reduces apoptosis in neural tissues, while significantly reducing hv*Kp* colonization in the central nervous system and multiple organs throughout the body, substantially prolonging the survival time of infected mice.

Of course, this study also has certain limitations. First, the experiment utilized the immortalized HCMEC/D3 cell line. Although this line serves as a standardized tool for studying the blood-brain barrier, it still differs from primary brain microvascular endothelial cells *in vivo* in terms of gene expression and function. Second, *in vivo*, the blood-brain barrier is a neurovascular unit composed of endothelial cells, pericytes, and astrocyte processes; this study has not yet addressed the effects of niacin on other components or intercellular interactions. Third, there is genetic and phenotypic heterogeneity among different clinical strains of hv*Kp*. This study utilized only a single standard highly virulent strain (ATCC 43816), and it remains to be verified whether the conclusions are applicable to other circulating strains. Fourth, this study used only male C57 mice and did not account for gender differences. Future studies will include mice of both sexes for further investigation.

In summary, this study systematically reveals for the first time the spatiotemporal dynamics of hv*Kp* -induced damage to HCMEC, characterized by “proliferation followed by apoptosis,” and elucidates that the activation of the oxidative stress-mediated ROS-p53-mitochondrial apoptosis pathway is the core mechanism by which hv*Kp* damages blood-brain barrier endothelial cells. Through *in vitro* and *in vivo* experiments, we systematically demonstrated that niacin at physiological concentrations can exert multiple effects—including endothelial protection, maintenance of the blood-brain barrier, and antagonism of central nervous system infection—by specifically regulating redox homeostasis, thereby providing a new therapeutic approach for the prevention and adjunctive treatment of hv*Kp* meningitis.

## Data Availability

The original contributions presented in the study are included in the article/supplementary material. Further inquiries can be directed to the corresponding author.
